# Potential of Visible and Near Infrared Spectroscopy and Pattern Recognition for Rapid Quantification of Notoginseng Powder with Adulterants

**DOI:** 10.3390/s131013820

**Published:** 2013-10-14

**Authors:** Pengcheng Nie, Di Wu, Da-Wen Sun, Fang Cao, Yidan Bao, Yong He

**Affiliations:** 1 College of Biosystems Engineering and Food Science, Zhejiang University, Hangzhou 310058, China; E-Mails: npc2012@zju.edu.cn (P.N.); china.di.wu@gmail.com (D.W.); caofang@zju.edu.cn (F.C.); ydbao@zju.edu.cn (Y.B.); 2 Food Refrigeration and Computerised Food Technology (FRCFT), Agriculture & Food Science Centre, School of Biosystems Engineering, University College Dublin, National University of Ireland, Belfield, Dublin 4, Ireland; E-Mail: dawen.sun@ucd.ie

**Keywords:** spectral analysis, adulteration, chemometrics, least-square support vector machine (LS-SVM), partial least square regression (PLSR), competitive adaptive reweighted sampling (CARS)

## Abstract

Notoginseng is a classical traditional Chinese medical herb, which is of high economic and medical value. Notoginseng powder (NP) could be easily adulterated with *Sophora flavescens* powder (SFP) or corn flour (CF), because of their similar tastes and appearances and much lower cost for these adulterants. The objective of this study is to quantify the NP content in adulterated NP by using a rapid and non-destructive visible and near infrared (Vis-NIR) spectroscopy method. Three wavelength ranges of visible spectra, short-wave near infrared spectra (SNIR) and long-wave near infrared spectra (LNIR) were separately used to establish the model based on two calibration methods of partial least square regression (PLSR) and least-squares support vector machines (LS-SVM), respectively. Competitive adaptive reweighted sampling (CARS) was conducted to identify the most important wavelengths/variables that had the greatest influence on the adulterant quantification throughout the whole wavelength range. The CARS-PLSR models based on LNIR were determined as the best models for the quantification of NP adulterated with SFP, CF, and their mixtures, in which the *r_P_* values were 0.940, 0.939, and 0.867 for the three models respectively. The research demonstrated the potential of the Vis-NIR spectroscopy technique for the rapid and non-destructive quantification of NP containing adulterants.

## 1. Introduction

Notoginseng the root of *Panax notoginseng* (also known as *Panax pseudoginseng*, or sanchi in Chinese), is a highly valued traditional Chinese medical plant because of its hemostatic and cardiovascular functions [1]. Notoginseng contains saponins (commonly referred to ginsenosides and notoginsenosides), essential oils, amino acids, polysaccharides, and flavonoids [2], and has been found to have pharmacological antioxidative, anti-inflammatory, anti-coagulation, neuroprotective, anti-fibrotic, anti-diabetic, anti-cancer, proangiogenic, cardiovascular and cerebrovascular ischemia protective functions, as well as anti-atherogenic effects [3].

Authentication of food and ingredients is of crucial concern to both consumers and food processors in public-health and economic terms. The purity of food ingredients is easily subject to abuse by suppliers [4,5]. Numerous food products are susceptible to being deliberately adulterated, especially when there are other low-cost products that have similar appearances and physical characteristics with the corresponding food products. Notoginseng is one such food product that is easily subject to tampering. Some businessmen deliberately adulterate notoginseng powder (NP) with *Sophora flavescens* powder (SFP) or corn flour (CF) into because of their much lower prices. Because SFP and CF have similar appearances and physical characteristics as NP, it is almost impossible for consumers to identify the purity of NP only by relying on naked eyes. At present, for most consumers, identification of NP mainly relies on the examiner's subjective senses [6].

Visible and near infrared (Vis-NIR) spectroscopy has been successfully proved as an efficient and advanced tool for rapid and nondestructive determination of food quality [7,8]. According to spectral ranges, Vis-NIR spectroscopy is generally divided into the visible spectrum (400–700 nm), short-wave NIR spectra (SNIR, 700–1,100 nm) and long-wave NIR spectra (LNIR, 1,100–2,500 nm). The visible spectrum is the portion of the electromagnetic spectrum that is visible to the human eye. It mainly records the color information of samples. NIR spectroscopy technique records the spectral bands that mainly correspond to C–H, O–H, and N–H vibrations, which are overtone and combination bands. Vis-NIR spectroscopy with fiber optic diffuse reflectance probe can be executed with little sample preparation and can be remotely controlled which makes the whole operation more convenient [9]. Vis-NIR spectroscopy has advantages over some of the conventional techniques of food analysis, e.g., it is rapid, timely and less expensive, hence is more efficient when a large number of samples are involved and many analyses are required. Moreover, Vis-NIR spectroscopy does not require expensive and time-consuming sample pre-processing or the use of chemical extractants. It is perhaps for these reasons that Vis-NIR spectroscopy could be considered as a possible alternative to enhance or replace conventional laboratory methods for the detection of NP adulterants. Recently, Vis-NIR spectroscopy is an emerging analytical technique to measure the internal qualities of powders [10–13]. Specifically in the analysis of the adulterant identification of powders,Wu, *et al.* [14] applied Vis-NIR spectroscopy for the rapid and noninvasive quantification of two common adulterants (flour and mungbean powder) in *Spirulina* powder. Borin, *et al.* [15] quantified common adulterants in powdered milk by NIR spectroscopy. Shi, *et al.* [16] applied NIR spectroscopy to characterize powder blending, testing a ternary powder mixture composed of lactose, avicel, and fine and coarse acetaminophen powder. However, to the best of our knowledge, no such research endeavors for the quantification of NP with adulterants using Vis-NIR spectroscopy technique have been reported yet.

Given the limited effort on the investigation of rapid techniques for determination NP with adulterants, the major objective of this study was to identify the feasibility of using Vis-NIR spectroscopic technique to rapidly and non-invasively quantify NP with adulterants. The specific aims of this paper were to: (i) quantify NP with adulterants using visible spectra (360–700 nm), SNIR spectra (700–1,040 nm) and LNIR spectra (937–2,500 nm) based on treatments with a single SFP adulterant, single CF adulterant, and the mixture of both adulterants; (ii) evaluate the adoption of partial least squares regression (PLSR) and least-squares support vector machines (LS-SVM) methods to accomplish the adulterant analysis; and (iii) select which spectral wavelengths may be best suited for the adulterant quantification.

## 2. Materials and Methods

### 2.1. Sample Preparation

Pure NP used in this study was obtained from Tongrentang Chinese Medicine (Beijing, China). The SFP used in this study was produced by Haozhou Daozhuang Co. Ltd., Haozhou, China. The CF used in this study was produced by Chengdu Hongsheng Co. Ltd., Chendu, China. Three NP sets were prepared: (1) a set of five NP treatments with SFP as a single adulterant; (2) a set of five NP treatments with CF as a single adulterant; and (3) a set of nine NP treatments with both SFP and CF as adulterants. The SFP constituents in the first NP treatment set were 0%, 5%, 10%, 15%, and 20% by mass (Design A in Table 1). Similarly, the CF constituents in the second NP treatment set were 0%, 5%, 10%, 15%, and 20% by mass (Design B in Table 1). Meanwhile, in the third NP treatment set, there were 5% and 5%, 5% and 10%, 5% and 15%, 10% and 5%, 10% and 10%, 10% and 15%, 15% and 5%, 15% and 10%, and 15% and 15% in percentages by mass for SFP and CF constituents, respectively (Design C in Table 1). Each treatment in any set had 20 samples, resulting in 100 samples in the treatment A and B sets, respectively, and 180 samples in treatment C set. All samples were prepared using an electronic balance. The mixing process was carried out using a mortar.

### 2.2. Spectral Measurement

A USB4000 Miniature Fiber Optic Spectrometer (The Ocean Optics, Inc., Dunedin, FL USA) was used to measure Vis-SNIR reflectance spectra of samples in the 350–1050 nm region. A NIR256-2.5 Spectrometer (The Ocean Optics, Inc) was used to measure LNIR reflectance spectra of samples in the range of 900–2,550 nm. Each sample had powders in a uniform container (1 cm in height, 1 cm in diameter). The surface of the sample was smoothed. A fiber-optic probe was placed at a distance of 10 mm and 90° angle away from the surface of the sample. The spectrum of each sample was the average of 10 successive scans. To improve the signal to noise ratio, spectra at some wavelengths were not considered. As a result, the spectra of wavelengths (360–700 nm) measured by USB4000 were used as the visible spectra (VIS), the spectra of wavelengths (700–1,040 nm) measured by USB4000 were used as the short-wave near infrared spectra (SNIR) and the spectra of wavelengths (937–2,500 nm) measured by NIR256-2.5 were used as the long-wave near infrared spectra (LNIR).

### 2.3. Model Calibration

In the model calibration, PLSR and LS-SVM were applied, respectively, to establish calibration models according to the spectral information of samples in the calibration set with their reference NP concentrations. After the model was established, the prediction set was then analyzed in order to estimate the actual predictive capability of the established models, to minimize the concrete risk of overfitting and to avoid chance correlations. The prediction set was independent of the calibration set and was applied only after the model was established. In this work, from the 20 samples in each treatment, 15 samples were used for calibration or model establishment, while the remaining five samples were used for prediction.

PLSR analysis proposed by Gerlach, *et al.* [17] is widely used for calibration in current spectral analyses methods. Known as a bilinear factor method, PLSR attempts to find multidimensional direction in the spectral matrix (X) that explains the maximum multidimensional variance direction in the column vector (Y; [18]. Both the spectra (response variables) and concentration (dependent variables) matrixes are decomposed simultaneously in the PLSR calculation. After the calculation, a set of orthogonal factors (latent variables, LVs) is projected. The first few LVs that are most related to predict dependent variables are then used for the model calibration. The calculation of PLSR was carried out using Unscrambler V9.7″ (CAMO PROCESS AS, Oslo, Norway).

LS-SVM is an least squares version of support vector machines (SVM) proposed by Suykens and Vandewalle [19]. It applies least squares error in the training error function [20]. LS-SVM finds the solution by solving a set of linear equations instead of a convex quadratic programming (QP) problem for classical SVM. Radial basis function (RBF) kernel was used as the kernel function of LS-SVM, as it is a nonlinear function and a more compact supported kernel [21]. A grid-search technique with leave one out cross-validation was used in the LS-SVM calibration process to determine the optimal parameter values of LS-SVM model, namely the regularization parameter γ and the RBF kernel function parameter σ2. For each combination of γ and σ2 parameters, the root mean square error of cross-validation (RMSECV) was calculated. The optimum parameters were selected when they produced the smallest RMSECV. The details of LS-SVM description was shown in the literature [22]. In this study, LS-SVM was executed using Matlab 2011a software (The Mathworks, Inc., Natick, MA, USA). The LS-SVM toolbox (LS-SVM v 1.5, Suykens, Leuven, Belgium) was applied in MATLAB to derive all of the LS-SVM models.

### 2.4. Variable Elimination Using Competitive Adaptive Reweighted Sampling (CARS)

Vis-NIR spectral data have a high degree of dimensionality with collinearity and redundancy among contiguous variables (wavelengths). Much of the same information is contained in the congruent wavelengths that are related to the similar constituents [23]. On the other hand, redundant information is included in those wavelength variables that are correlated with their neighboring variables. Moreover, some variables may contain irrelevant information or noise rather than pertinent information to quality attributes of samples. Eliminating those collinear and redundant variables from the full-spectrum has shown positive improvements on the prediction accuracy in many cases [24–27]. In this study, CARS was used to select the most important variables that had less redundancy and contributed most in the quantification of adulterated NP. CARS algorithm was proposed by Li, *et al.* [28] to select an optimal combination of the variables from the full range variables coupled with PLSR. The selection is based on the absolute coefficients of variables in the PLSR model, which are set as an index for evaluating the importance of each variable. The variables with large absolute coefficients have more chance to be selected in the CARS calculation. In general, there are four successive steps in each CARS sampling run, namely Monte Carlo model sampling, enforced wavelength reduction by exponentially decreasing function (EDF), competitive wavelength reduction by adaptive reweighted sampling (ARS) and RMSECV calculation for each subset. Monte Carlo (MC) sampling runs aim to select the variables that are of high adaptability regardless of the variation of training samples. EDF is used to eliminate the variables with relatively small absolute regression coefficients. ARS is carried out to further select variables utilizing the principle of ‘survival of the fittest’ that is the basis of Darwin's Evolution Theory [29]. At last, the optimal variable set is determined according to the RMSECV. In this work, the processes of CARS selection were performed with the aid of Matlab 2011a software. The model establishment using the full range spectra (328 variables for visible spectra, 378 variables for SNIR, and 241 variables for LNIR) was called Method I; while using only the important wavelengths selected by CARS was called Method II throughout this paper.

### 2.5. Model Evaluation Standard

The predictive abilities of the models were evaluated according to some statistics, such as correlation coefficient of calibration (*r_C_*), root mean square error of calibration (RMSEC) and coefficient of determination of calibration ( $R_{C}^{2}$) for the calibration process, and correlation coefficient of prediction (*r_P_*), root mean square error of prediction (RMSEP), residual predictive deviation (RPD), and coefficient of determination of prediction ( $R_{P}^{2}$) for the prediction process. The standard for evaluating the performance of a model is that a good model should have high correlation coefficients (*r_C_* and *r_P_*), high coefficient of determination ( $R_{C}^{2}$ and $R_{P}^{2}$), and the low root mean square errors (RMSEC and RMSEP) as well as a small difference between RMSEC and RMSEP.

## 3. Results and Discussion

### 3.1. Spectral Analysis

**Figure 1.**
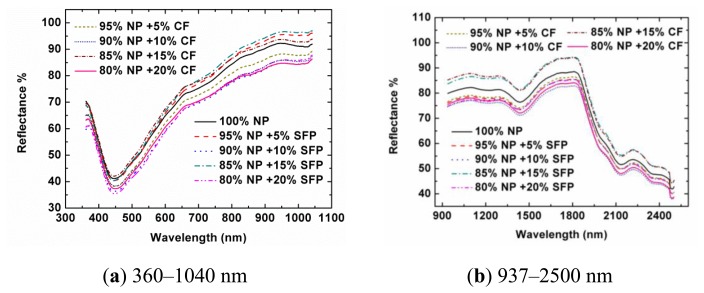
Spectral patterns of the tested notoginseng powder (NP) adulterated by different concentrations of sophora flavescens powder (SFP) and/or corn flour (CF) in 360–1,040 nm (**a**) and 937–2,500 nm (**b**). Percentages are shown by mass (g/g).

Figure 1 shows the spectra of samples from Designs A and B in Vis-NIR regions. In the visible region, there were absorption peaks for all the curves around 450 nm, and the spectra at other bands were generally reflected. This was the reason why the pure NP and adulterated NP had a grey colour. In the SNIR region, a weak absorbance was found around 980 nm that was assigned to the O–H stretching second overtone of water, which was explainable as there was little water in the NP. The absorbance at 1,225 nm was assigned to the second overtone of C–H stretching. The absorbance at 1,450 nm was assigned to the first overtone of O–H stretching. The absorbance at 2,140 nm was assigned to the combination overtone of C–H and C=C stretching. The absorbance at 2,380 nm was assigned to the second overtone of O=C deformation. The absorbance at 2,488 nm was assigned to the combination overtone of C–H and C–C stretching of starch [30,31]. Generally speaking, the spectral profiles of samples from Designs A and B had similar trends and appearances, respectively. In the visible region, that was mainly because the colors of SFP and CF were very close to that of NP. In the near-infrared region, it was also difficult to observe the differences between the pure samples and the adulterated samples. There were three main reasons for this: firstly, in the adulterated samples, the contents of SFP and CF in the samples were relatively low. Secondly, these three kinds of powders (NP, SFP, and CF) are all organic matters and have similar chemical bonds, resulting in similar spectral profiles. Thirdly, the near-infrared spectra are overtone and combination bands of the mid-infrared spectra. There were many wide absorption bands with overlaps, weak absorption and low sensitivity for the pure and adulterated NP samples. There was no feature peak directly related to the adulterants in the reflectance spectral profiles or the second derivative spectra (data are not shown). When more samples were considered, the spectral profiles of the tested samples showed various magnitudes. Therefore, the adulteration couldn't be directly discriminated from the spectra only by naked eyes. It was still difficult to find relationships between spectra and the content of NP directly. Instead, chemometrics were employed for the data mining and analysis. Because most spectral preprocessing algorithms are conducted based on the full range spectra and it is difficult to obtain the preprocessed spectra at only several optimal wavelengths by using wavelength dispersion devices, no preprocessing treatments were applied to the spectral data during the selection of optimal wavelengths and the development of the calibration model in this study.

### 3.2. Quantitation of Adulterated NP Based on Full Range Spectra

Establishment of regression models for the quantification of NP adulterated by SFP (Design A) CF (Design B), and the mixture of two adulterants (Design C) was executed using LS-SVM algorithm based on the data of visible, SNIR, and LNIR spectra, respectively (Table 2). When visible spectra were used for the model establishment, the LS-SVM model had good prediction for Design A. The statistical result expressed as *r_C_* between the samples' full range spectra and their NP concentrations was 0.971 with a RMSEC of 1.693%. In addition, the model had a *r_P_* of 0.932 and a RMSEP of 2.778%. When Design B was analyzed, the LS-SVM of visible spectra had a reasonable result with a *r_C_* of 0.950 and a RMSEC of 2.269 in calibration, and a *r_P_* of 0.845, and a RMSEP of 3.809% in prediction. Meanwhile, the performance of the LS-SVM model based on visible spectra was not satisfactory for Design C, where *r_P_* was only 0.688 and RMSEP was over 4%. When SNIR was used for the model establishment, the LS-SVM model had good results for both Designs A and B. In general, the SNIR had similar prediction result to the visible spectra for Design A, but had better result for Design B, where the RMSEP decreased by 53.7% to 2.029%. However, the SNIR also failed for Design C. Its LS-SVM model had the RMSEP of 3.830 with *r_P_* of 0.786. When LNIR was applied, similar results were obtained for Designs A and B, compared with the visible and SNIR spectra. On the other hand, the LNIR offered a good prediction for Design C, in which the *r_C_* was 0.892, the *r_P_* was 0.898, and the RMSEP was less than 2%. PLSR was also considered for the model calibration. In most cases, LS-SVM obtained better results than PLSR, except the case that LNIR was used for Design B. In general, the analysis of Designs A and B could be successfully achieved by using visible or SNIR spectra, which were both acquired using the USB4000 Miniature Fiber Optic Spectrometer. However, due to the limited information on hydrogen containing bonds, such as O–H, C–H, and N–H provided by visible and SNIR spectra, it was difficult to use the spectra from 360 nm to 1,040 nm to do the quantification of NP adulterated by the mixture of two adulterants. Because more information relevant to the hydrogen-containing bonds is contained in the LNIR, it showed its extraordinary capability of prediction compared to the visible and SNIR spectra for Design C. In addition, by analyzing the absolute differences between RMSECV and RMSEP, which was a standard to evaluate the robustness of established models, it was found that only the LS-SVM model established based on SNIR for Design C was overfitted, where the difference was over 3%. Other models had their differences less than 2%, showing that the most LS-SVM models (Method I) for the quantification of adulterated NP were not overfitted and had a good robust feature.

### 3.3. Identification of Effective Wavelengths Using CARS

**Figure 2.**
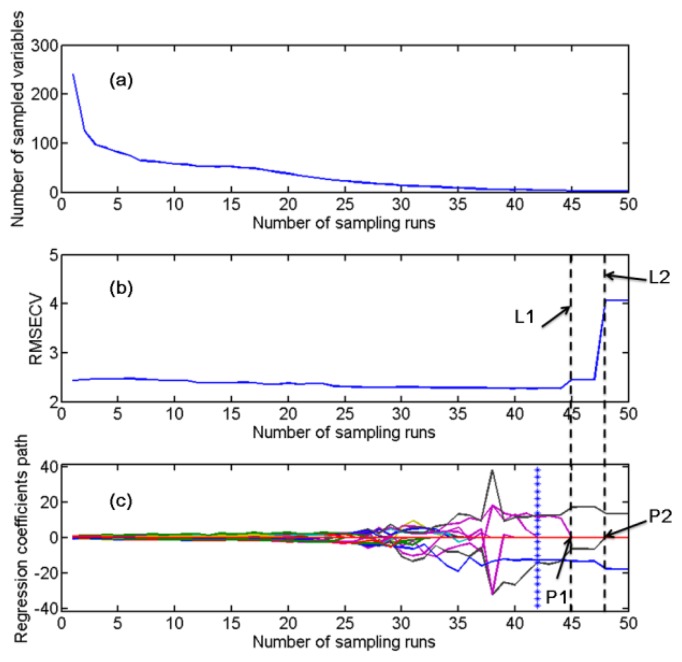
Changing trends of the number of sampled variables in the competitive adaptive reweighted sampling (CARS) calculation. (**a**) 5-fold the root mean square error of cross-validation (RMSECV) values; (**b**) and regression coefficients of each variable; (**c**) with the increasing of sampling runs. The line (marked by asterisk) denotes the optimal point where 5-fold RMSECV values achieve the lowest.

CARS was carried out to select the effective variables by using the simple but effective principle “survival of the fittest” on which Darwin's Evolution Theory is based. The CARS calculation was executed based on the visible spectra, SNIR, and LNIR, respectively. As an example, the variation trends of some key parameters in CARS along with the increment of sampling runs based on the analysis of the LNIR spectra of samples in the calibration set for Design B are shown in Figure 2, in which there are three sub-figures included. Figure 2a shows the variation trend of the number of sampled variables during the calculation. After a stepwise selection of CARS, only effective variables were kept while other insignificant variables were removed efficiently. Figure 2b shows the tendency of 5-fold RMSECV values along with the increase in the number of sampling runs. Despite that there was no much change of RMSECV before the 45th run, the variable number dramatically decreased during the calculation. The RMSECV reached the smallest value of 2.268%, when the run times reached 42, which was denoted by an asterisk line. Only four variables remained at this step. After that, the RMSECV increased abruptly in two phases due to the removing of two informative variables, proving that these two variables were important to the model calibration. The model's prediction ability would be reduced dramatically without considering these variables. One of the variables is indicated by P1 in Figure 2c. When it was eliminated as its coefficient dropped to zero, the RMSECV rose up as indicated by dot line L1. Another case is that the coefficient of another variable denoted by P2 dropped to zero, resulting in the sharp rising of RMSECV value denoted by dot line L2. The principle of CARS calculation could be understood more visualized by analyzing the regression coefficient path of each variable (Figure 2c). Each variable had its own regression coefficient path during the CARS calculation. When they were removed by CARS, their coefficients dropped to zero, which is somewhat like the incompetence species are exterminated. The remained variables with large coefficients would get more probability to survive, just like the ‘survival of the fittest’ in Darwin's Evolution Theory. After the CARS calculation, an optimal combination of some competent wavelengths was retained with uninformative variables eliminated. As a result of the CARS calculation, there were eight, three, and six variables selected as the effective variables for visible spectra, SNIR, and LNIR respectively in Design A, eleven, six, and four variables in Design B, and four, four, and eight variables in Design C. The specific effective variables selected by CARS for visible spectra, SNIR, and LNIR for the quantification of NP adulterated by SFP, CF and the mixture of two adulterants are shown in Table 3.

### 3.4. Quantitation of Adulterated NP Using Selected Wavelengths

As a consequence of the variable selection, new reduced spectral matrix was generated by selecting the spectral data only at the effective variables that contained the most relevant spectral information of adulteration detection. The new matrix was then used to replace the full range spectra for building new quantification models. In order to choose the optimal calibration method for the adulteration quantification, the performances of two calibration algorithms of PLSR and LS-SVM were compared based on the selected variables. Table 4 shows the results of regression models for the quantification of NP adulterated by SFP, CF and the mixture of two adulterants based on the selected wavelengths. When visible spectra were used for the model calibration, good predictions were obtained by the CARS-LS-SVM models for Designs A and B with an average *r_P_* of 0.921 and an average RMSEP of 2.868%. The CARS-PLSR model obtained a similar result to the CARS-LS-SVM model for Design A, but its prediction for Design B was not as good as for Design A. Both CARS-LS-SVM and CARS-PLSR models failed for Design C, in which their RMSEP were larger than 4%. In general, the results of variable selection were acceptable for visible spectra. After most variables eliminated, the performances of the CARS-LS-SVM models maintained the same levels of the corresponding LS-SVM models (Method I).

When SNIR spectra were considered, the CARS-LS-SVM models did good prediction for Designs A and B with an average *r_P_* of 0.942 and an average RMSEP of 2.372%, which were similar to the corresponding LS-SVM models (Method I). On the other hand, the quantification of adulterated NP in Design C was still not successful with the RMSEP over 4%, when the CARS-LS-SVM model was established based on SNIR. Moreover, the CARS-LS-SVM model was still overfitted with the absolute differences between RMSECV and RMSEP over 2%. However, it was noticed that the difference of the LS-SVM model after variable selection (Method II) was much reduced from 3.275% to 2.130%, compared with the LS-SVM model (Method I). CARS-PLSR models were also established based on SNIR. However, their performances were worse than the corresponding CARS-LS-SVM models for all three designs. Especially for Design B, the RMSEP of the CARS-PLSR model was over twice as much as that of the CARS-LS-SVM model. By comparing the results of LS-SVM models (Method I) and the CARS-LS-SVM models for SNIR, it was found that the variable selection could remain the performances for Designs A and B, but was not very successful for Design C, where the RPD value decreased by 11.48%. When the spectra were switched from SNIR to LNIR, both the CARS-PLSR and CARS-LS-SVM models had the *r_C_* and *r_P_* higher than 0.9, showing their good prediction for Designs A and B. Moreover, it was noticed that different from the visible and SNIR spectra, the LNIR could detect the quantification of adulterated NP for the Design C based on either the CARS-PLSR model or the CARS-LS-SVM model. This result was similar to that of the quantitation of adulterated NP based on full range spectra of LNIR.

According to model evaluation standard, the performances of the CARS-PLSR and CARS-LS-SVM models were compared. For the Design A, the best model was determined as the CARS-LS-SVM model of LNIR, which had the *r_C_* of 0.979 and *r_P_* of 0.953. The RPD value of this model was 3.198, which means the model is usable for most applications [32]. Similarly, the best models for the Designs B and C were determined as the CARS-LS-SVM models of SNIR and LNIR, respectively. Their RPD values were 3.624 and 2.152, indicating they were usable for screening and for most applications, respectively [32]. On the other hand, the optimal models for adulteration detection should not only consider the model's accuracy, but also the convenience of the model establishment. In general, the establishment of PLSR models does not need the projection of kernel functions, and therefore is simpler than that of LS-SVM models. When only the CARS-PLSR models were considered, the best prediction models were all determined as the LNIR's models for three designs. Actually the performances of CARS-PLSR models of LNIR were acceptable. The models' RPD values for the Designs A and B were almost 2.9 and that for the Design C was almost 2. The above results show that the LNIR spectroscopy was the most suitable one among the three spectral ranges for the quantification of NP with adulterants. Its measurement of using a small spectrometer (dimensions of 153.4 × 105.2 × 76.2 mm) was nondestructive, inexpensive, and very convenient to implement. The CARS-PLSR functions of LNIR for Designs A, B, and C are shown as follows:

(1) $$\left[Y_{\text{Design A}}=135.7+29.3X_{937\;\text{nm}}-24.1X_{984\;\text{nm}}-11.5X_{1508\;\text{nm}}+13.8X_{1951\;\text{nm}}-10.5X_{2003\;\text{nm}}+4.1X_{2407\;\text{nm}}\right]$$

(2) $$\left[Y_{\text{Design B}}=96.9-8.3X_{1580\;\text{nm}}+3.2X_{1886\;\text{nm}}+16.2X_{1945\;\text{nm}}-13.4X_{2311\;\text{nm}}\right]$$

(3) $$\left[Y_{\text{Design C}}=9.5-13.0X_{944\;\text{nm}}+5.1X_{1004\;\text{nm}}+7.6X_{1018\;\text{nm}}-9.3X_{1606\;\text{nm}}+17.1X_{1912\;\text{nm}}-7.4X_{2048\;\text{nm}}-6.1X_{2496\;\text{nm}}+4.7X_{2502\;\text{nm}}\right]$$

In general, the quantifications of adulterated NP for Designs A and B were easier than that for Design C. The appreciable decline in the performances results for Design C compared with Designs A and B was most likely caused by the simultaneous presence in the NP samples of two adulterants of SFP and CF whose components likely produced complexity in spectra, making the use of visible and SNIR spectra incompatible. Given LNIR's richer information on hydrogen containing bonds than other two spectral regions, it was not surprising that the LNIR spectra performed better. The LNIR spectroscopy technique provided adequate quantitation of NP samples adulterated with not only one adulterant of SFP or CF but also their mixture.

## 4. Conclusions

The potential of Vis-NIR spectroscopy for rapid quantification of NP with adulterants was investigated. The results discussed in this paper indicated that the LNIR spectroscopy was satisfactory as a rapid and convenient tool for assessing the NP concentration. Three wavelength ranges of visible, SNIR and LNIR were separately considered for the model establishment, and their performances were compared. The results show that the all three ranges could do the NP quantification efficiently when one adulterant of SFP (Design A) or CF (Design B) was added into NP. On the other hand, when both SFP and CF were added into NP (Design C), only the LNIR spectra, which contained more spectral information on hydrogen containing bonds, obtained a good prediction on NP concentration, while the visible and SNIR spectra failed in this case. By means of CARS algorithm, a few important spectral variables were selected from the full range spectra, so that the high dimensionality with redundancy and collinearity among the Vis-NIR spectra was reduced. Moreover, there was a general lowering of the difference between RMSEP and RMSEC for CARS models, showing that they were more robust than those models established using the full range spectra. Considering both the model's accuracy and the convenience of the model establishment, the best quantitative models for Designs A, B, C were all determined as the CARS-PLSR models with LNIR. In view of the adulterant detection of NP, the results of this study verified the substantial propensity of the Vis-NIR spectroscopic technology to be an excellent alternative to the time-consuming and laborious processes.

## Figures and Tables

**Table 1.** Sets of five notoginseng powder (NP) treatments with a single adulterant of *Sophora flavescens* powder (SFP) (Experimental design A), with a single adulterant of corn flour (CF) (Experimental design B), and with both SFP and CF as adulterants (Experimental design C).

**Experimental Design**	**Treatment No.**	**NP% by Mass (g/g)**	**SFP% by Mass (g/g)**	**CF% by Mass (g/g)**	**Sample Number**
A	1	100	0	0	20
	2	95	5	0	20
	3	90	10	0	20
	4	85	15	0	20
	5	80	20	0	20

B	1	100	0	0	20
	2	95	0	5	20
	3	90	0	10	20
	4	85	0	15	20
	5	80	0	20	20

C	1	90	5	5	20
	2	85	5	10	20
	3	80	5	15	20
	4	85	10	5	20
	5	80	10	10	20
	6	75	10	15	20
	7	80	15	5	20
	8	75	15	10	20
	9	70	15	15	20

**Table 2.** Results of regression models for the quantification of Notoginseng powder (NP) adulterated by sophora flavescens powder (SFP), corn flour (CF), and the mixture of two adulterants using least-squares support vector machines (LS-SVM) algorithm based on the data of visible spectra, short-wave near infrared spectra (SNIR), and long-wave near infrared spectra (LNIR), respectively.

**Adulterant**	**Spectral Range**	**Modeling Method**	**LVs**	**Calibration**			**Prediction**				
	
				***r_C_***	$R_{C}^{2}$	**RMSEC (%)**	***r_P_***	$R_{P}^{2}$	**RMSEP (%)**	**RPD**	
SFP	Visible	LS-SVM	/	0.971	0.943	1.693	0.932	0.846	2.778	2.670
		PLSR	6	0.939	0.882	2.427	0.911	0.787	3.261	2.314
	SNIR	LS-SVM	/	0.961	0.922	1.972	0.921	0.841	2.815	2.559
		PLSR	4	0.867	0.751	3.528	0.874	0.762	3.451	2.055
	LNIR	LS-SVM	/	0.984	0.967	1.276	0.917	0.840	2.829	2.501
		PLSR	4	0.924	0.853	2.709	0.912	0.821	2.994	2.362
CF	Visible	LS-SVM	/	0.950	0.897	2.269	0.845	0.710	3.809	1.858
		PLSR	10	0.936	0.876	2.489	0.792	0.602	4.461	1.623
	SNIR	LS-SVM	/	0.994	0.987	0.796	0.959	0.918	2.029	3.514
		PLSR	5	0.851	0.724	3.716	0.821	0.670	4.062	1.746
	LNIR	LS-SVM	/	0.986	0.973	1.166	0.930	0.864	2.609	2.724
		PLSR	5	0.957	0.916	2.047	0.946	0.893	2.308	3.071
SFP&CF	Visible	LS-SVM	/	0.834	0.685	3.241	0.688	0.471	4.198	1.376
		PLSR	2	0.548	0.300	4.830	0.574	0.327	4.735	1.220
	SNIR	LS-SVM	/	0.996	0.991	0.555	0.786	0.560	3.830	1.577
		PLSR	1	0.576	0.332	4.720	0.571	0.325	4.743	1.218
	LNIR	LS-SVM	/	0.892	0.794	2.621	0.898	0.789	2.652	2.183
		PLSR	8	0.887	0.787	2.665	0.871	0.754	2.862	2.033

LVs: Number of latent variables.

**Table 3.** Selected effective variables by competitive adaptive reweighted sampling (CARS) for visible spectra, short-wave near infrared spectra (SNIR), and long-wave near infrared spectra (LNIR) for the quantification of notoginseng powder (NP) adulterated by *Sophora flavescens* powder (SFP), corn flour (CF) and the mixture of two adulterants, respectively.

**Adulterant**	**Spectral Range**	**Selected Effective Variables (nm)**
SFP	Visible	406, 408, 431, 439, 475, 476, 537, 697
	SNIR	755, 926, 1016
	LNIR	937, 984, 1508, 1951, 2003, 2407
CF	Visible	506, 508, 509, 511, 541, 578, 579, 621, 629, 634, 699
	SNIR	700, 750, 865, 980, 992, 1040
	LNIR	1580, 1886, 1945, 2311
SFP&CF	Visible	361, 393, 699, 700
	SNIR	737, 745, 858, 941
	LNIR	944, 1004, 1018, 1606, 1912, 2048, 2496, 2502

**Table 4.** Results of regression models for the quantification of notoginseng powder (NP) adulterated by *Sophora flavescens* powder (SFP), corn flour (CF), and the mixture of two adulterants using partial least squares regression (PLSR) and least-squares support vector machines (LS-SVM) algorithm based on the spectra at the competitive adaptive reweighted sampling (CARS) selected wavelengths of visible spectra, short-wave near infrared spectra (SNIR), and long-wave near infrared spectra (LNIR), respectively.

**Adulterant**	**Range**	**NV^1^**	**Modeling Method**	**LVs^2^**	**Calibration**			**Prediction**			
	
					***r****_C_*	$R_{C}^{2}$	**RMSEC (%)**	***r****_P_*	$R_{P}^{2}$	**RMSEP (%)**	**RPD**
SFP	Visible	8	LS-SVM	/	0.966	0.933	1.836	0.918	0.822	2.979	2.376
	Visible	8	PLSR	5	0.956	0.914	2.072	0.914	0.822	2.982	2.375
	SNIR	3	LS-SVM	/	0.924	0.854	2.700	0.922	0.846	2.779	2.555
	SNIR	3	PLSR	3	0.718	0.515	4.923	0.864	0.741	3.598	1.967
	LNIR	6	LS-SVM	/	0.979	0.959	1.431	0.953	0.894	2.305	3.198
	LNIR	6	PLSR	5	0.953	0.909	2.136	0.940	0.878	2.466	2.869
CF	Visible	11	LS-SVM	/	0.961	0.924	1.949	0.923	0.848	2.757	2.581
	Visible	11	PLSR	7	0.893	0.797	3.183	0.816	0.639	4.250	1.703
	SNIR	6	LS-SVM	/	0.964	0.928	1.897	0.963	0.923	1.964	3.624
	SNIR	6	PLSR	5	0.854	0.729	3.684	0.805	0.628	4.314	1.646
	LNIR	4	LS-SVM	/	0.987	0.974	1.136	0.949	0.898	2.253	3.139
	LNIR	4	PLSR	3	0.954	0.910	2.124	0.939	0.880	2.453	2.899
SFP&CF	Visible	4	LS-SVM	/	0.744	0.542	3.907	0.664	0.433	4.348	1.328
	Visible	4	PLSR	3	0.563	0.317	4.773	0.579	0.324	4.746	1.219
	SNIR	4	LS-SVM	/	0.929	0.857	2.180	0.709	0.443	4.310	1.411
	SNIR	4	PLSR	3	0.649	0.421	4.392	0.620	0.382	4.540	1.274
	LNIR	8	LS-SVM	/	0.903	0.815	2.481	0.891	0.781	2.704	2.152
	LNIR	8	PLSR	6	0.881	0.777	2.729	0.867	0.744	2.921	1.998

1. NV= Number of variables;

2. LVs= Number of latent variables.
